# Parenting Style Dimensions As Predictors of Adolescent Antisocial Behavior

**DOI:** 10.3389/fpsyg.2016.01383

**Published:** 2016-09-13

**Authors:** David Álvarez-García, Trinidad García, Alejandra Barreiro-Collazo, Alejandra Dobarro, Ángela Antúnez

**Affiliations:** Department of Psychology, University of OviedoOviedo, Spain

**Keywords:** family, parenting style, antisocial behavior, adolescence, evaluation

## Abstract

Antisocial behavior is strongly associated with academic failure in adolescence. There is a solid body of evidence that points to parenting style as one of its main predictors. The objective of this work is to elaborate a reduced, valid, and reliable version of the questionnaire by Oliva et al. (2007) to evaluate the dimensions of parenting style and to analyze its psychometric properties in a sample of Spanish adolescents. To that end, the designed questionnaire was applied to 1974 adolescents 12–18 years of age from Asturias (Spain). Regarding construct validity, the results show that the model that best represents the data is composed of six dimensions of parenting style, just as in the original scale, namely affection and communication; promotion of autonomy; behavioral control; psychological control; self-disclosure; and humor. The psychological control factor negatively correlates with the other factors, with the exception of behavioral control, with which it positively correlates. The remaining correlations among the factors in the parenting style questionnaire are positive. Regarding internal consistency, the reliability analysis for each factor supports the suitability of this six-factor model. With regard to criterion validity, as expected based on the evidence available, the six dimensions of parenting style correlate in a statistically significant manner with the three antisocial behavior measures used as criteria (off-line school aggression, antisocial behavior, and antisocial friendships). Specifically, all dimensions negatively correlate with the three variables, except for psychological control. In the latter case, the correlation is positive. The theoretical and practical implications of these results are discussed.

## Introduction

Antisocial behavior, which is defined as behavior that violates social norms and the rights of others (Burt, 2012), constitutes an important problem in adolescence. Regarding the most serious, identified, and proven cases of antisocial behavior, the rate of minors between 14 and 17 of age in Spain who were convicted in 2014 is 8.7 per 1000 (National Statistical Institute [Instituto Nacional de Estadística], 2015). However, it is reasonable to assume that the prevalence of antisocial behavior among young people, although difficult to specify, is greater than the data indicate. This type of behavior causes significant personal and social damage. Those who engage in antisocial behavior considerably reduce their educational and employment opportunities; those who suffer it must endure its physical, emotional, or economic consequences. In the social sphere, these problems consume a large amount of resources related to mental health, education, and juvenile justice (Sawyer et al., 2015).

Among the various factors identified as predictors of antisocial behavior in adolescence, the type of educational and relational practices exercised by parents stand out (Álvarez-García et al., 2015; Cutrín et al., 2015). Although these types of practices may vary in different situations, relatively stable attitudes and behavioral patterns with specific effects on the behavior of children can be identified. These practices are called “parenting styles” (Torío et al., 2008). One of the most commonly used typologies of parenting style is that proposed by MacCoby and Martin (1983) based on a reformulation of the work by Baumrind (1967). This classification distinguishes four types of parenting styles, based on two dimensions (*responsiveness/acceptance* and *demandingness/control*): authoritative (responsive and demanding); indulgent (responsive but not demanding); authoritarian (demanding but not responsive); and neglectful (neither responsive nor demanding).

Previous research offers consistent results regarding the existence of a significant association between parenting style and antisocial behavior in adolescents. Thus, parental practices characterized by affection, communication, and support (responsiveness) are negatively associated with antisocial behavior in children, including drug use (García and Gracia, 2009; Pérez, 2012; Calafat et al., 2014), criminal behavior (García and Gracia, 2009; Ginsburg et al., 2009; Hoeve et al., 2011), inconsiderate and disrespectful treatment of parents (Pérez, 2012), behavioral problems in school (García and Gracia, 2009), and bullying (Kokkinos, 2013; Gómez-Ortiz et al., 2015). These studies point to the neglectful parenting style (low responsiveness and demandingness) as that which is most positively associated with antisocial behavior in adolescents.

Although the role of responsiveness (affection, communication, and support) in child behavior seems to be clear, that of demandingness is less clear. Its effect on general adolescent behavior, particularly on the adolescent’s possible antisocial behavior depends on the type of demandingness exercised by parents. In general terms, positive and negative demandingness can be differentiated (Alegre, 2011). Positive demandingness involves parental practices that include the parent’s reasoned guidance of children on desirable behavior, empathetic explanations, behavior monitoring, the promotion of autonomy in children, and demands and expectations according to children’s degree of maturity. By contrast, negative demandingness involves parental practices that include psychological control that hampers child autonomy through behaviors such as excessive control, emotional blackmail, and the withdrawal of affection and attention or guilt induction if the child does not do what is asked, in addition to punitive (screams, punishments, and threats) and severe discipline. Negative parental demandingness, compared to positive demandingness, is associated with an increased likelihood of internalized and externalized problems and with less emotional competence in children (Alegre, 2011).

Because all of these nuances must be taken into account, more dimensions than the two basic dimensions proposed by MacCoby and Martin (1983) are being considered to define the different types of parenting styles. A good example is the six-dimension model proposed by Oliva et al. (2008). In this model, four parental control-related variables are considered: *behavioral control* (establishing behavioral boundaries and monitoring activities, friendships, and places frequented by the children when the parents are not present); *self-disclosure* (a subtle form of control consisting of children’s spontaneous disclosure to their parents of what they do in their free time, typically resulting from an affective and communicative bond between parents and children); *psychological control* (the parental use of manipulative strategies, including guilt induction or emotional blackmail); and the *promotion of autonomy* (the parental stimulation of children’s freedom and independence in decision-making processes related to the problems that affect them). The two remaining variables relate to family communication: *affection* (parental attitudes that include listening, supporting, and understanding their children) and *humor* (a relaxed, cheerful, and optimistic parental attitude). From the scores obtained by parents in these six dimensions, Oliva et al. (2008) distinguish three types of parents: democratic, strict, and indifferent.

The diversity of models regarding parenting dimensions and styles has given rise to a variety of instruments to assess them. One of these instruments is the parenting style questionnaire proposed by Oliva et al. (2007), based on the six-dimension model by Oliva et al. (2008). This questionnaire has been chosen by other researchers to analyze the relationship between parenting styles and various aspects in adolescents including reading comprehension development (Carpio et al., 2012), academic failure (Sabán et al., 2013), pregnancy risk (Pérez-López et al., 2015), psychopathological symptoms (Rosa-Alcázar et al., 2014), resilience (Gómez-Ortiz et al., 2015), child-on-parent violence (Calvete et al., 2014), and involvement in bullying (Gómez-Ortiz et al., 2014). The previous studies that use this questionnaire to analyze the relationship between its dimensions and antisocial behavior in adolescents found that affection and communication, promotion of autonomy, behavioral control, and humor perceived by adolescents in their parents and self-disclosure reported by adolescents correlate negatively with external problems and substance abuse (Oliva et al., 2007), and hostility (Rosa-Alcázar et al., 2014); while parental psychological control perceived by adolescents correlate positively with these antisocial behaviors. Calvete et al. (2014), using only the affection and communication factor, found that this dimension is a significant protective factor of both physical and psychological aggression against parents.

The parenting style questionnaire by Oliva et al. (2007) has shown its theoretical and practical utility in the various studies in which it has been used. It is based on a solid theoretical model, and contrasted with the validation test, it displays adequate psychometric properties. It has helped identify the parenting style of the parents evaluated and analyze the relationship of each style with the behavior of children. However, one possible problem with its application can be its length. It consists of 82 items, which, when applied within a battery of tests and particularly when applied to younger people, can be problematic. Developing an abbreviated version of the test and checking whether it can provide researchers with a valid and reliable measure of the parenting style dimensions that is sufficiently informative and useful for their research purposes would be of great interest.

For all of these reasons, the objective of this work is to elaborate a reduced, valid, and reliable version of the questionnaire to evaluate the parenting style dimensions proposed by Oliva et al. (2007) and to analyze its psychometric properties in a sample of Spanish adolescents. Shortening the test is not expected to adversely affect the validity and reliability of its measurements.

## Materials and Methods

### Participants

A total of 2045 adolescents from 10 schools participated in the study. They were selected through stratified random sampling from all schools in Asturias (Spain) supported with public funds that provide Compulsory Secondary Education (Educación Secundaria Obligatoria – ESO). Schools supported with public funds constitute 95.9% of the schools that provide ESO in Asturias. To select the sample, the schools were divided according to their ownership (public or semi-private), and in each stratum, a number of schools proportionate to the population were selected. In Spain, public schools are those in which both their management and funding are entirely public, and semi-private schools (*centros concertados*) are those with private management but partial public financing. This stratification variable was used as previous studies suggest that public and semi-private schools in Asturias differ in the socioeconomic status of families and students’ academic performance (Fernández and Muñiz, 2012). As a result, six public and four semi-private schools were selected. All students under ESO at each school were evaluated.

Once samples with a significant number of blank or void responses were discarded, the final sample comprised 1974 adolescents between 12 and 18 years of age (mean = 14.02; *SD* = 1.38). A total of 49.1% were girls; 28.1% of the students evaluated are in their first year, 25.4% in their second year, 25.0% in their third year, and 21.5% in their fourth year.

### Measurement Instruments

#### Parenting Style

An adaptation of the parenting style scale by Oliva et al. (2007) was developed. The original scale measured six parental dimensions: affection and communication; promotion of autonomy; behavioral control; psychological control; self-disclosure; and humor. To that end, adolescents must respond to 41 items regarding their father’s parenting style and 41 regarding their mother’s parenting style (82 total). The response format is a six-point Likert-type scale. The adapted version, used in the present work, introduces three modifications to the original scale: after a pilot test, the number of items was reduced from 41 to 24 (four per factor), once factor loadings and item correlations were analyzed; the subject is asked to jointly assess both parents’ parenting style, if he or she has two parents (therefore, the subject answers only 24 items); and the response options are reduced from six to four (1 = completely false; 2 = somewhat false; 3 = somewhat true; 4 = completely true). The students had to indicate the extent to which each assertion in the scale was true. The final questionnaire applied to the students is shown in the Appendix.

#### Off-Line School Aggression

A self-report scale, which was designed and previously used by the research team, was used in this study (Álvarez-García et al., 2016). It has six items involving the frequency with which the subject expressed having behaved aggressively in the physical school environment over the last 3 months: “No he dejado participar en mi grupo a algún compañero, durante alguna actividad de recreo o de Educación Física” [“I excluded some of my classmates from interacting in my group, during some leisure activity or in Physical Education class”], “No he dejado participar en mi grupo a algún compañero en alguna actividad de clase” [“I excluded some of my classmates from participating in some class activities in my group”], “Me he reído y burlado de algún/a compañero/a” [“I laughed at and made fun of a classmate”], “He hablado mal de algún/a compañero/a a sus espaldas” [“I spoke ill of some classmates behind their backs”], “He insultado a la cara a algún/a compañero/a” [“I insulted some of my classmates to their face”], and “He pegado a algún/a alumno/a del centro, dentro o a la salida del recinto escolar” [“I hit a student in school or when leaving school grounds”]. The response is a four-point Likert-type scale (1 = never; 2 = a few times; 3 = many times; 4 = always). The internal consistency of the scale in the sample for this study is high (α = 0.84).

#### Antisocial Behavior

A scale developed *ad hoc* for this study was used adapting some items from the “Antisocial and criminal behavior scale in adolescents” by Andreu and Peña (2013). The scale used consists of six items: “He ensuciado, dañado o destruido conscientemente mobiliario público (por ej., una pared, una papelera, una farola, asientos del autobús)” [“I consciously soiled, damaged, or destroyed public furniture (e.g., a wall, a trashcan, a lamppost, seats on the bus)”], “He robado algo de una tienda, del colegio o de una casa” [“I stole something from a shop, school, or a private home”], “He entrado sin permiso en una propiedad privada” [“I trespassed on private property”], “He golpeado o me he peleado con un desconocido hasta dañarle” [“I have hit or fought with a stranger to the point of harming him/her”], “He consumido drogas ilegales” [“I used illegal drugs”], and “Me he emborrachado” [“I have gotten drunk”]. The requested response is dichotomous (true/false), stating whether the subject has performed these actions over the last year. The internal consistency of the scale in this sample is acceptable (KR20 = 0.73).

#### Antisocial Friendships

A scale developed *ad hoc* for this study was used. Inspired by some of the indicators of antisocial behavior proposed by Andreu and Peña (2013), it is composed of four items: “Alguno/a de mis mejores amigos/as ha ensuciado, dañado o destruido conscientemente mobiliario público (por ej., una pared, una papelera, una farola, asientos del autobús)” [“One or some of my best friends have soiled, damaged, or destroyed public furniture (e.g., a wall, a trashcan, a lamppost, seats on the bus)],” “Alguno/a de mis mejores amigos/as ha robado algo de una tienda, del colegio o de casa” [“One or some of my best friends have stolen something from a shop, school, or a private home”], “Alguno/a de mis mejores amigos/as se ha peleado físicamente en serio con otro/a chico/a” [“One or some of my best friends have had a real physical fight with another young person”], and “Alguno/a de mis mejores amigos/as ha consumido drogas ilegales” [“One or some of my best friends have consumed illegal drugs”]. The requested response is dichotomous (true/false), stating whether the subject has performed these actions over the last year. The internal consistency of the scale in this sample is acceptable (KR20 = 0.71).

### Procedure

First, the questionnaires used in the study were selected or designed. Subsequently, 10 schools, whose students constitute the study sample, were selected. Then, permission to apply the questionnaires was requested from the schools’ respective head management teams. Each management team was informed of the objectives and procedures of the study, its voluntary and anonymous nature, and the confidential treatment of the results. Once the schools agreed to participate, informed consent was requested from the parents or guardians of students because the students are minors. Before answering the questionnaire, the students were also informed of the anonymous, confidential, and voluntary nature of their participation. In general, the students had 20 min to complete the questionnaires, although timing was flexible depending on the age and characteristics of the subjects. The test was applied by the investigating team to all groups in each school during school hours.

### Data Analysis

The factorial validity of the scores from the parenting style questionnaire was analyzed using the EQS 6.2 statistical program (Bentler, 2014). Although not severe, given the non-normality of the data and the ordinal nature of the scale, the robust maximum likelihood estimating method was used, and the analyses were conducted based on the polychoric correlations matrix (Hoyle, 2012). Questionnaires with three or more blank or null items were removed (71). To avoid losing more samples and to be able to use all available data, the missing values were treated by computing the covariance matrix through the pairwise method.

To determine the degree of fit of the models tested, the Satorra-Bentler scaled chi-square (SBχ^2^)/degrees of freedom (df), the robust comparative fit index (RCFI), the robust Bentler-Bonett non-normed fit index (RNNFI), the root mean square error of approximation (RMSEA), and the robust Akaike information criterion (RAIC) were used. Typically, values indicative of a good fit are CFI ≥ 0.95, NNFI ≥ 0.95, and RMSEA ≤ 0.06 (Hu and Bentler, 1999), and χ^2^/df < 3 (Ruiz et al., 2010). The RAIC makes it possible to compare models, and that with the lowest value is preferable.

Once the model with the best fit to the data was identified, its discriminant validity was studied by analyzing the correlation between its factors and each item’s factorial weight. Very high correlations (*r* ≥ 0.85) warn of potential collinearity or redundancy among factors, thus pointing to poor discriminant validity (Brown, 2015). Factorial weights above 0.30 are typically considered acceptable (Izquierdo et al., 2014).

Reliability for each subscale was analyzed in terms of internal consistency; each subscale’s Cronbach alpha coefficient, from the polychoric correlations matrix, was found. The squared multiple correlation of each item was estimated to indicate the variance proportion in the item explained by the latent variable, thus calculating each item’s reliability to measure the variable (Bollen, 1989).

Finally, SPSS 21 (IBM Corp, 2012) statistical software was used to analyze criterion validity. To that end, the Spearman coefficient of correlation between the score in each of the six factors in the parenting style questionnaire and the three external criteria was calculated. Regarding the three measures used as criteria, namely, off-line school aggression, antisocial behavior, and antisocial friendships, there is evidence of their association with parenting style. The score in each of these three factors was obtained by adding the scores for each of the items that compose them.

## Results

### Construct Validity

The goodness of fit of the 6FM (six-factor model; the model that best fitted the data in the validation study of the original questionnaire) was tested with the reduced version of the scale, which was designed and administered in the present study. Subsequently, its fit was compared with that of another model that was also plausible from a theoretical perspective. This alternative model, composed of two factors [2FM (two-factor model)], corresponds to the classical two-dimension distinction that defines parenting styles: affection and control (**Table 1**). In both models, the factors are latent variables that are significantly related to each other and free from error of measurement; each item (observable indicator) is explained only by a factor and is associated with a certain error of measurement. The results obtained show that the 6FM is the model that best fits the empirical data obtained (**Table 2**).

**Table 1:** Proposed models to analyze the dimensionality of the reduced parenting style questionnaire.

Model	Factors	Items
2FM	Responsiveness/acceptance	1, 2, 3, 4, 21, 22, 23, and 24
	Demandingness/control	5, 6, 7, 8, 9, 10, 11, 12, 13, 14, 15, 16, 17, 18, 19, and 20
6FM	Affection and communication	1, 2, 3, and 4
	Promotion of autonomy	5, 6, 7, and 8
	Behavioral control	9, 10, 11, and 12
	Psychological control	13, 14, 15, and 16
	Self-disclosure	17, 18, 19, and 20
	Humor	21, 22, 23, and 24

*2FM, two-factor model; *6FM*, six-factor model.*

**Table 2:** Goodness-of-fit indexes of the two models tested for the reduced parenting style questionnaire with the total sample (*N* = 1974).

Model	*SB*χ*^2^*	*df*	*p*	*SB*χ*^2^/df*	RCFI	RNNFI	RMSEA (CI 90%)	RAIC
2FM	7437.56	251	<0.001	29.63	0.869	0.856	0.120 (0.118–0.123)	6935.56
6FM	838.33	237	<0.001	3.54	0.989	0.987	0.036 (0.033–0.038)	364.33

*2FM, two-factor model; *6FM*, six-factor model; *SBχ^*2*^*, Satorra-Bentler Scaled Chi-Square; *df*, degrees of freedom; *p*, probability; *RCFI*, robust comparative fit index; *RNNFI*, robust non-normed fit index; *RMSEA*, root mean square error of approximation; *CI*, confidence interval; *RAIC*, robust Akaike information criterion.*

**FIGURE 1:**
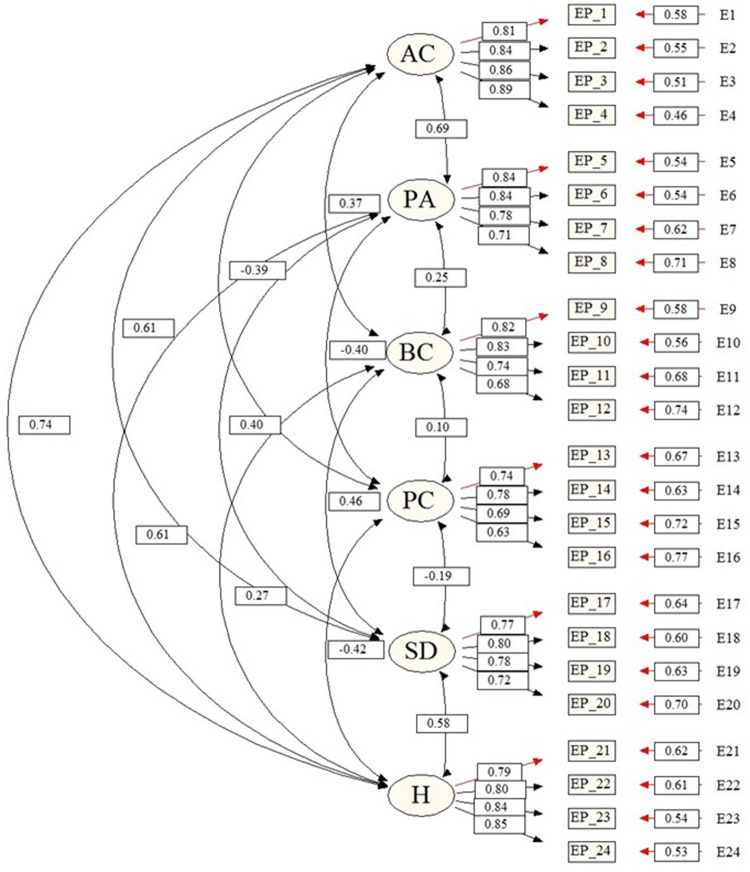
**Factorial structure of the reduced parenting style questionnaire (AC, affection and communication; PA, promotion of autonomy; BC, behavioral control; PC, psychological control; SD, self-disclosure; H, humor)**.

As shown in **Figure 1**, the psychological control factor negatively correlates with the other factors. The only exception is behavioral control, with which it positively correlated. The other correlations among the factors are positive. None of the correlations among the factors is greater than 0.85. The strongest correlation is found between the affection and communication factor and the humor factor (*r* = 0.74), and the weakest correlation is found between behavioral control and psychological control (*r* = 0.10). All correlations are significant.

The factorial weights of each item in its factor generally present high values (**Figure 1**). In 21 of the 24 items, the standardized regression coefficient is greater than 0.70. The three exceptions (items 12, 15, and 16) present values higher than 0.60.

### Reliability

The internal consistency of the scores for each factor is high. The Cronbach’s alpha coefficient is greater than 0.80 for all factors (**Table 3**). Nonetheless, redundant items do not appear: the polychoric correlations between items of the same factor present values between 0.70 and 0.82 for affection and communication; between 0.56 and 0.79 for promotion of autonomy; between 0.52 and 0.71 for behavioral control; between 0.45 and 0.61 for psychological control; between 0.53 and 0.65 for self-disclosure; and between 0.64 and 0.76 for humor. Item reliability (*R^2^*) is moderate or high. The proportion of item variance explained by the latent variable is between 40.0 and 79.1% (**Table 3**).

**Table 3:** Reliability for each factor and item in the reduced parenting style questionnaire (*N* = 1974).

Factor	Item	α	*R*^2^
Affection and communication		0.925	
	1		0.663
	2		0.700
	3		0.744
	4		0.791
Promotion of autonomy		0.878	
	5		0.705
	6		0.709
	7		0.611
	8		0.501
Behavioral control		0.854	
	9		0.667
	10		0.689
	11		0.542
	12		0.457
Psychological control		0.806	
	13		0.554
	14		0.609
	15		0.478
	16		0.400
Self-disclosure		0.859	
	17		0.593
	18		0.639
	19		0.607
	20		0.517
Humor		0.902	
	21		0.621
	22		0.633
	23		0.710
	24		0.717

*α, Cronbach’s alpha coefficient; R^2^, Squared multiple correlation.*

### Criterion Validity

The scores obtained in each of the six factors of the reduced parenting style questionnaire significantly correlate with the scores in each of the three external criteria analyzed: off-line school aggression, antisocial behavior, and antisocial friendships (**Table 4**). Psychological control positively correlates with these three variables. The other five factors of the parenting style questionnaire negatively correlate with them. The magnitude of the correlation coefficients is, in general terms, weak.

**Table 4:** Spearman’s correlation coefficient between the score in each factor of the reduced parenting style questionnaire and scores in the scales: off-line school aggression, antisocial behavior, and antisocial friendships (*N* = 1974).

	Off-line school aggression	Antisocial behavior	Antisocial friendships
Affection and communication	-0.19*	-0.22*	-0.20*
Promotion of autonomy	-0.14*	-0.08*	-0.10*
Behavioral control	-0.11*	-0.21*	-0.14*
Psychological control	0.15*	0.14*	0.18*
Self-disclosure	-0.26*	-0.34*	-0.29*
Humor	-0.19*	-0.16*	-0.16*

*p ≤ 0.001.

## Discussion

The objective of this work was to elaborate a reduced, valid, and reliable version of the questionnaire by Oliva et al. (2007) to evaluate the dimensions of parenting style and to analyze its psychometric properties in a sample of Spanish adolescents. As initially hypothesized, the results obtained show that shortening the test does not adversely affect the validity and reliability of its measurements, presenting suitable metric properties to be administered with the purpose for which it was designed.

Regarding construct validity, the 6FM proposed by Oliva et al. (2007) has shown a good fit to the data obtained when applying the short version designed in the present work. The fit indexes obtained are even better than those obtained by its creators in the validation of the original scale (Oliva et al., 2007). The factorial weight for each item in its factor is high, generally higher than those obtained by Oliva et al. (2007).

The six dimensions of parenting style measured by the questionnaire significantly correlate with each other, with a moderate magnitude. This result supports the discriminant validity of the scores obtained: although the six dimensions are related, they have sufficient entity to be considered distinct constructs. The correlations between factors are not sufficiently high to be considered redundant. In general, the pattern of results is very similar to that obtained by Oliva et al. (2007) when validating the original questionnaire. On the one hand, higher correlations are found among affection and communication, humor, promotion of autonomy, and self-disclosure. On the other hand, psychological control negatively correlates with the other factors with the exception of behavioral control, with which it positively correlates. The remaining correlations among factors are positive. These results suggest some important issues. First, although promotion of autonomy and self-disclosure are concerned with parental control, they are closely related to affection and communication and humor, as originally predicted. Although the methodology used does not make it possible to establish causal relationships, prior longitudinal studies (Kearney and Bussey, 2015) suggest that close and optimistic parents may promote greater autonomy in children and create an atmosphere in which adolescents feel confident to spontaneously tell their parents what they have done or how they feel. Second, the results of this study suggest that disclosure is more likely to occur in a context in which adolescents perceive that their parents are interested in what happens to them (behavioral control) and that it is less likely to occur in a context in which the use of excessive control or manipulative strategies (psychological control) is perceived. Previous studies combining transverse and longitudinal analysis coincide with the present work in finding a significant cross association between these variables, although the results of the longitudinal analysis cast doubt on the causal nature of the relationship between control and disclosure (Kearney and Bussey, 2015). This aspect must be further investigated in the future. Third, psychological control, defined as excessive control and the use of manipulative strategies, relates to parental practices characterized by little affection and communication, humor, and promotion of autonomy. Psychological control only positively correlates with behavioral control, most likely because both aim at establishing behavioral boundaries, although the strategies for achieving them are different. However, the correlation between behavioral control and psychological control is the weakest among those found between factors in this study.

The reliability analysis of the scores obtained with the test supports the relevance of the 6FM. The internal consistency for the scores in each factor is high. The alpha values obtained for each factor are similar and in some cases even higher than those found in the original scale (Oliva et al., 2007; Sabán et al., 2013; Calvete et al., 2014; Rosa-Alcázar et al., 2014; Gómez-Ortiz et al., 2015). Therefore, reducing the items by factor to four does not seem to have negatively affected factor internal consistency. Item reliability is also high, suggesting that the observable indicators used are good descriptors of the questionnaire dimensions.

With regard to criterion validity, as expected based on the evidence available, the six dimensions of parenting style present a significant association with the three antisocial behavior measures used as criteria (off-line school aggression, antisocial behavior, and antisocial friendships). In particular, the results obtained show that the greater the affection and communication, promotion of autonomy, behavioral control, and humor perceived by adolescents in their parents and the greater the self-disclosure reported by adolescents, the lower the off-line school aggression, antisocial behavior, and antisocial friendships recognized by adolescents. By contrast, the greater the parental psychological control perceived by adolescents is, the greater the off-line school aggression, antisocial behavior, and antisocial friendships recognized by them. Self-disclosure is the dimension that is most closely related to these three antisocial behavior variables. These results are consistent with previous studies that use the original version of the parenting style questionnaire by Oliva et al. (2007): the same pattern of results regarding the external problems and substance abuse variables (Oliva et al., 2007) and hostility (Rosa-Alcázar et al., 2014) variables is found. Calvete et al. (2014) use only the affection and communication factor in their study, finding that it is a significant protective factor of both physical and psychological aggression against fathers and mothers. As in the present study, correlations magnitude in previous research is commonly weak, due to the existence of additional variables, different from the parenting style dimensions analyzed, that also affect the emergence of antisocial behavior in adolescence (Slattery and Meyers, 2014).

Other studies that have analyzed the relationship between parenting styles and antisocial behavior in adolescence using different assessment instruments have obtained similar results. As indicated in the Introduction, parental practices characterized by affection, communication, and support are negatively associated with antisocial behavior in children, including drug use (García and Gracia, 2009; Pérez, 2012; Calafat et al., 2014), criminal behavior (García and Gracia, 2009; Ginsburg et al., 2009; Hoeve et al., 2011), inconsiderate and disrespectful treatment of their parents (Pérez, 2012), behavioral problems in school (García and Gracia, 2009), and active involvement in bullying (Kokkinos, 2013; Gómez-Ortiz et al., 2015).

In this study, the parenting style dimension most closely associated with low levels of antisocial behavior is self-disclosure. This variable stands out in previous studies as a control method that is potentially more effective in the prevention of antisocial behavior than active parental methods of control (asking questions). In these studies, parent-child closeness is positively related to self-disclosure by adolescents. In turn, self-disclosure positively relates to parental awareness of what children do, and this knowledge is negatively related to antisocial behavior in children (Soenens et al., 2006; Vieno et al., 2009).

Regarding psychological control – the only parental dimension positively associated with antisocial behavior in children – previous meta-analysis studies have emphasized that this variable is a significant predictor of delinquency, even greater than behavioral control (Hoeve et al., 2009). The negative correlation between psychological control and self-disclosure can be found among the varied mechanisms that may explain the relationship between psychological control and delinquency. This negative correlation may affect parental awareness regarding children’s behavior, in addition to substance abuse and delinquency (Soenens et al., 2006). Obtaining results that are consistent with prior available evidence supports the criterion validity of the test.

The present work has various theoretical and practical implications. From a theoretical perspective, the results support the relevance of the six-dimension model of parenting styles considered in the original scale. The dimensions considered have an entity of their own, although they are interrelated, and the observable indicators used are good descriptors of the construct evaluated. From a practical perspective, a brief questionnaire with sufficient metric guarantees for the evaluation of six fundamental dimensions to identify the parenting style from the perspective of the adolescent is made available to researchers and professionals in psychology. The relationship between the parenting style dimensions and the three external criteria observed in this study supports the importance of taking into account parenting styles in the prevention and treatment of antisocial behavior in adolescents. Affection and communication with children constitute an essential variable in preventing and treating this type of behavior.

For all of these reasons, the present work represents a contribution to the study of the relationship between parenting styles and antisocial behavior in adolescents. However, it also presents some limitations. First, the developed and validated short questionnaire does not allow one to distinguish certain aspects related to family context that may affect adolescents’ social behavior, including the type of family structure (Breivik and Olweus, 2006), the shared parenting style by both parents (Berkien et al., 2012), and which style in the couple has a greater effect on adolescent behavior (Tur-Porcar et al., 2012). The original questionnaire asked adolescents to evaluate their fathers and mothers separately. By contrast, the abbreviated questionnaire forces adolescents to decide which is the predominant style in their family. Previous studies using the original version of the questionnaire show moderate-to-high correlations between the father and the mother in each factor (between 0.61 and 0.85 in Gómez-Ortiz et al., 2015; between 0.46 and 0.79 for girls and between 0.57 and 0.84 for boys in Oliva et al., 2007). In addition, the metric properties of both versions were almost identical in the validation by Oliva et al. (2007). However, testing the reduced version of the scale for each member of the couple separately would be appropriate in the future due to its practical utility. Second, readers should bear in mind that a significant percentage of young people with antisocial behavior live in shelters or have completely dysfunctional families, with a lack of parental figures and constant changes in guardianship (Orrego et al., 2016). Therefore, this questionnaire would not apply in these cases, although the evaluator should record this circumstance. Third, the questionnaire has been validated with a random and broad sample of adolescents but a sample that is limited to some ages and a specific geographical context. Therefore, any generalization of the study results to other ages and contexts should be made with caution. In the future, validating this scale in other ages and contexts would be of interest. Fourth, the relationship between the questionnaire dimensions and the three antisocial behavior variables was analyzed using a correlational methodology. Thus, the results obtained in this study do not make it possible to establish causal relationships between the variables analyzed. Although the review of evidence has made it possible to refine these results, it would be interesting to use this questionnaire in longitudinal studies in the future. Fifth, some of the questionnaires used were designed *ad hoc* for the present study; thus, they were not previously validated in other samples. Sixth and finally, in analyzing the relationship between parenting style and antisocial behavior, the role of other potentially relevant variables, such as the socioeconomic situation of the family, which can be an important stressor, has not been taken into account.

## Author Contributions

DA-G: Designed the study, analyzed the data, and wrote the manuscript. TG: Analyzed the data and wrote the manuscript. AB-C, AD, and AA: Recruited the subjects, collected data, and contributed to interpretation of data and critical revision of the article.

## Conflict of Interest Statement

The authors declare that the research was conducted in the absence of any commercial or financial relationships that could be construed as a potential conflict of interest.
