# Supplementary material for: Parenting Style Dimensions As Predictors of Adolescent Antisocial Behavior
Source: Front Psychol. 2016 Sep 13;7:1383. doi: 10.3389/fpsyg.2016.01383 (PMC5020069; doi:10.3389/fpsyg.2016.01383)
Supplement: Supplementary file 1 [file Data_Sheet_1.DOCX]

**Appendix**

Please indicate the extent to which the following statements are true:

|  | 1 | 2 | 3 | 4 |
| --- | --- | --- | --- | --- |
| 1. Cuando hablo con mis padres, muestran interés y atención [When I speak with my parents, they show interest and pay attention]. |  |  |  |  |
| 2. Mis padres me animan a que les cuente mis problemas y preocupaciones [My parents encourage me to tell them about my problems and concerns]. |  |  |  |  |
| 3. Si tengo algún problema puedo contar con la ayuda de mis padres [If I have a problem, I can count on my parents’ help]. |  |  |  |  |
| 4. Mis padres muestran interés por mí cuando estoy triste y enfadado/a [My parents show concern when I am sad and angry]. |  |  |  |  |
| 5. Mis padres piensan que aunque aún no sea una persona adulta puedo tener ideas acertadas [My parents think that even though I am not an adult yet, I can have good ideas]. |  |  |  |  |
| 6. Mis padres me animan a que tome mis propias decisiones [My parents encourage me to make my own decisions]. |  |  |  |  |
| 7. Mis padres me animan a que piense de forma independiente [My parents encourage me to think independently]. |  |  |  |  |
| 8. Mis padres me permiten opinar cuando hay que tomar una decisión familiar [My parents allow me to express my opinion when making a family decision]. |  |  |  |  |
| 9. Mis padres intentan saber a dónde voy cuando salgo [My parents try to find out where I am going when I leave home]. |  |  |  |  |
| 10. Si vuelvo tarde a casa, mis padres me preguntan por qué y con quién estuve [If I return home late, my parents ask me why I was late and who I was with]. |  |  |  |  |
| 11. Mis padres ponen límites a la hora a la que debo volver a casa [My parents set a curfew for me]. |  |  |  |  |
| 12. Mis padres me preguntan en qué gasto el dinero [My parents ask me how I spend money]. |  |  |  |  |
| 13. Mis padres me hacen sentir culpable cuando no hago lo que quieren [My parents make me feel guilty when I do not do what they want]. |  |  |  |  |
| 14. Mis padres me dicen que ellos tienen razón y no debo llevarles la contraria [My parents tell me that they are right and that I must not contradict them]. |  |  |  |  |
| 15. Mis padres intentan controlar continuamente mi forma de ser y de pensar [My parents continuously try to monitor the way I am and think]. |  |  |  |  |
| 16. Mis padres dejan de hablarme cuando se enfadan conmigo [My parents stop talking to me when they get angry at me]. |  |  |  |  |
| 17. Les cuento a mis padres lo que hago en mi tiempo libre [I tell my parents what I do in my free time]. |  |  |  |  |
| 18. Les hablo a mis padres sobre los problemas que tengo con mis amigos/as [I speak to my parents about the problems I have with my friends]. |  |  |  |  |
| 19. Cuando llego de la escuela, le cuento a mis padres cómo me ha ido el día [When I get home from school, I tell my parents about my day]. |  |  |  |  |
| 20. Aunque no me pregunten, les cuento a mis padres cómo me va en las diferentes asignaturas [Although they do not ask me, I tell my parents how I am doing in my different classes at school]. |  |  |  |  |
| 21. Mis padres casi siempre son personas alegres y optimistas [My parents are almost always cheerful and optimistic people]. |  |  |  |  |
| 22. Mis padres suelen bromear conmigo [My parents tend to make jokes with me]. |  |  |  |  |
| 23. Es divertido hacer cosas con mis padres [It is fun to do things with my parents]. |  |  |  |  |
| 24. Mis padres se ríen mucho conmigo [My parents laugh a lot with me]. |  |  |  |  |
| 1 = completely false; 2 = somewhat false; 3 = somewhat true; 4 = completely true | | | | |
